# History of Mind Cure

**Published:** 1888-05

**Authors:** Helen Densmore


### HISTORY OF MIND CURE.

BY DR. HELEN DENSMORE.

[The following is taken from the first numbers of Earnest Words, and explains itself :]

THERE is a most remarkable phenomenon visable in this country at the present time in a wide-spread belief in what is called Mind Cure, Mental Healing, Christian Science, and various other appellations, but all meaning the same thing : the power of the mind to cure physical ailments. It is comparatively a new "craze," although Mrs. Eddy, the founder of the new school—so far as its introduction to the world as a formulated system is concerned—has been teaching it in Boston for over twenty years. It is only within the past three years that it has attracted public attention and permeated the social atmosphere ; now, one hears of it in nearly every circle into which he is introduced. If we inquire into it, we find various schools with rival claims, notwithstanding the fact that they all seem to have originated in and in essential principle and method of demonstration, are off-shoots and repetitions of Mrs. Eddy's teaching. Although dissensions and misunderstandings have divided them into various and oft-times hostile camps, their teaching is fundamentally the same. This underlying doctrine is held by them all, to wit : there is no such thing as sickness, sin and death ; that these things are the result of erroneous judments, false mental states, and exist in the mind alone. There is no physiology, no organic process, no functional activity. These are all modes of mental activity ; all is in the mind ; matter has no existence save as an idea in mind. Muscular force is mental force ; all functional action, prehension, deglutition, digestion, assimilation, and excretion are performed by thought ; in short, there is no phisiology ; and all pain, suffering, sin, and death are in the world because of a belief in the mind that they exist. A proper understanding of the truth dispels the error, and the suffering disappears. All disease is but a reflection of thought, and has no existence in reality. It is only a photograph of distorted thoughts which have taken the place of perfect ones; and which would in place of the distortion give us a picture of health, without reference to material conditions of any kind.

All this being very metaphysical, brings us back to Berkley and his philosophy, and we would be inclined in this utilitarian age of steam and electricity to relegate it all to the incomprehensible realm but for the fact that a decidedly practical side to the matter appears in the marvelous cures that are related, and which are daily being verified.

This new school of healing has no sympathy with Spiritualism or Theosophy; it is considered in no way a gift, but an understanding of a truth which to comprehend is all that is required. It bears no relation to faith cures; requires, it is said, neither faith on the part of the patient, nor peculiar conditions of sympathy between the patient and the healer.

It is claimed as a science, and we find all about us classes being taught by teachers representing the various schools; and from these classes are being constantly sent out healers who report their marvelous cures. The course usually consists of twelve lessons, and the price of tuition for the course ranges from $300 to $25. Mrs. Eddy charges $300, and her graduates charge as a rule $100. Among the first of Mrs. Eddy's pupils were several women who, refusing to accept her personal dictum, branched out and became themselves heads of schools. Of this number Mrs. Newman and Mrs. Stuart, of Boston, have had remarkable success in both teaching and demonstrating the new science. They have, by their personal influence, ability, and force of character, carried the radical medical reform into the heart of conservatism; and the anathemas of scientists and spiritual heads of the church alike fail to prevent it.

If we take the present indication as true prophecy, the mental mystics are fast moving on Materia Medica, entrenched behind centuries of practice with pill, potion, and physic. Our large cities have become centres of propaganda. New York, Boston, Chicago, and San Francisco have each several colleges, and their literature has already assumed surprising proportions.

I propose to give in the following pages these lessons in full, as given by Mrs. Eddy, Mrs. Stuart, and Mrs. Newman; to point out the teachings of the various schools; and to follow with a statement showing wherein this system, *as formulated and now taught*, although containing a great and important truth, is destined to do more injury in encouraging license in the matter of diet, in ignoring the physiological nature of man, and in teaching contempt for hygiene and an orderly physiological life, than all the good accomplished by its phenomenal cures.

In succeeding numbers Mrs. Densmore gave a synopsis of the teachings of Mrs. Eddy and of her book called Science and Health: also of the lessons as given by Mrs. Stuart. We have arranged with the author for the publication in our Journal of the completion of this series of articles. Since, as was explained in *Earnest Words*, the teachings of all the schools of this so-called science are based upon Mrs. Eddy's work and as they are substantially the same it has been deemed unnecessary to republish the articles devoted to Mrs. Eddy and Mrs. Stuart—since the lessons of Mrs. Newman which we will give in our next number cover the whole ground.

